# Extreme Precipitation and Emergency Room Visits for Gastrointestinal Illness in Areas with and without Combined Sewer Systems: An Analysis of Massachusetts Data, 2003–2007

**DOI:** 10.1289/ehp.1408971

**Published:** 2015-04-09

**Authors:** Jyotsna S. Jagai, Quanlin Li, Shiliang Wang, Kyle P. Messier, Timothy J. Wade, Elizabeth D. Hilborn

**Affiliations:** 1 Division of Environmental and Occupational Health Sciences, School of Public Health, University of Illinois, Chicago, Illinois, USA; 2 National Health and Environmental Effects Research Laboratory, Office of Research and Development, U.S. Environmental Protection Agency, Research Triangle Park, North Carolina, USA; 3 Biostatistics and Bioinformatics Research Center, Samuel Oschin Comprehensive Cancer Institute, Cedars Sinai Medical Center, Los Angeles, California, USA; 4 Department of Environmental Science and Engineering, UNC Gillings School of Global Public Health, Chapel Hill, North Carolina, USA

## Abstract

**Background:**

Combined sewer overflows (CSOs) occur in combined sewer systems when sewage and stormwater runoff are released into water bodies, potentially contaminating water sources. CSOs are often caused by heavy precipitation and are expected to increase with increasing extreme precipitation associated with climate change.

**Objectives:**

The aim of this study was to assess whether the association between heavy rainfall and rate of emergency room (ER) visits for gastrointestinal (GI) illness differed in the presence of CSOs.

**Methods:**

For the study period 2003–2007, time series of daily rate of ER visits for GI illness and meteorological data were organized for three exposure regions: *a*) CSOs impacting drinking water sources, *b*) CSOs impacting recreational waters, *c*) no CSOs. A distributed lag Poisson regression assessed cumulative effects for an 8-day lag period following heavy (≥ 90th and ≥ 95th percentile) and extreme (≥ 99th percentile) precipitation events, controlling for temperature and long-term time trends.

**Results:**

The association between extreme rainfall and rate of ER visits for GI illness differed among regions. Only the region with drinking water exposed to CSOs demonstrated a significant increased cumulative risk for rate (CRR) of ER visits for GI for all ages in the 8-day period following extreme rainfall: CRR: 1.13 (95% CI: 1.00, 1.28) compared with no rainfall.

**Conclusions:**

The rate of ER visits for GI illness was associated with extreme precipitation in the area with CSO discharges to a drinking water source. Our findings suggest an increased risk for GI illness among consumers whose drinking water source may be impacted by CSOs after extreme precipitation.

**Citation:**

Jagai JS, Li Q, Wang S, Messier KP, Wade TJ, Hilborn ED. 2015. Extreme precipitation and emergency room visits for gastrointestinal illness in areas with and without combined sewer systems: an analysis of Massachusetts data, 2003–2007. Environ Health Perspect 123:873–879; http://dx.doi.org/10.1289/ehp.1408971

## Introduction

Climate change is expected to bring changes in the frequency and severity of weather events such as precipitation, flooding, and hurricanes, which can impact human health (Easterling et al. 2000; Ebi et al. 2006; Patz et al. 2001, 2005; Rose et al. 2001). Waterborne disease outbreaks are preceded by heavy precipitation events in the United States (Curriero et al. 2001) and the United Kingdom (Nichols et al. 2009), and extreme precipitation was linked to waterborne infections in Taiwan (Chen et al. 2012). Typical seasonal variations in rainfall and temperature have been associated with specific gastrointestinal (GI) diseases including cholera (Checkley et al. 2000; Olago et al. 2007; Pascual et al. 2000), cryptosporidiosis (Jagai et al. 2009), and rotavirus (Cook et al. 1990; Jagai et al. 2012; Levy et al. 2009). Acute GI illness in children has been associated with rainfall 4 days prior (Drayna et al. 2010), and heavy rainfall was associated with increased risk of hospitalization for GI illness (Bush et al. 2014).

Areas with combined sewer systems and aging infrastructure are particularly vulnerable to adverse water quality impacts resulting from increased precipitation events (Levin et al. 2002; Patz et al. 2008). Modern systems have separate collection for storm water and sewage, but many urban areas in the United States have combined sewer systems that collect rainwater runoff, domestic sewage, and industrial waste together, in one pipe, for transport to wastewater treatment facilities. During periods of heavy rainfall, the volume of wastewater can exceed system capacity and is discharged directly to nearby streams, rivers, or other water bodies before treatment, resulting in a combined sewer overflow (CSO). CSO events can contaminate water sources with pathogenic microorganisms associated with untreated sewage, including protozoa, viruses, and bacteria such as *Cryptosporidium* sp., *Salmonella* sp*.,* and norovirus (Donovan et al. 2008; Levin et al. 2002; Marsalek and Rochfort 2004). The largest waterborne disease outbreak in the United States, which occurred in Milwaukee, Wisconsin, in 1993, is thought to have been due to passage of cryptosporidium oocysts through the filtration system of a water treatment plant following heavy rainfall that impaired source water quality (MacKenzie et al. 1995).

The U.S. Environmental Protection Agency (EPA) states that 772 communities of about 40 million people total, primarily in the Northeast, Great Lakes area, and the Pacific Northwest, are served by combined sewer systems (U.S. EPA 2008). A control policy was established in 1994 to establish a protocol for reporting discharges through the National Pollutant Discharge Elimination System (U.S. EPA 1994). In addition, communities are expected to develop long-term CSO control plans to attain water quality standards compliant with the Clean Water Act (U.S. EPA 1995).

Studies have demonstrated that pathogen concentrations in receiving waters are higher following CSO events. A bacterial indicator of fecal contamination, *Escherichia coli*, was increased in recreational waters in Ontario, Canada (Marsalek and Rochfort 2004) and Lake Michigan (McLellan and Salmore 2003) following CSO events. Increases in concentrations of bacteria associated with sewage, including *Streptococcus*, *Enterococcus*, and several pathogenic viruses, were seen following CSO events in the Lower Passaic River (Donovan et al. 2008). Given the potential for increased CSO events due to changes in precipitation patterns associated with climate change, it is important to better understand impacts on human health.

The goal of this study was to assess whether the association between heavy rainfall and rate of emergency room (ER) visits for GI illness differed in the presence or absence of CSO systems. The study used data from the state of Massachusetts, which has 24 permitted combined sewer systems located primarily in the central and eastern part of the state (Massachusetts Executive Office of Energy and Environmental Affairs 2014). We hypothesized that heavy precipitation (≥ 90th and 95th percentiles) and, especially, extreme precipitation (≥ 99th percentile) would be associated with an increased rate of ER visits for GI illness in areas with CSOs compared with areas without CSOs.

## Methods

**Figure 1:**
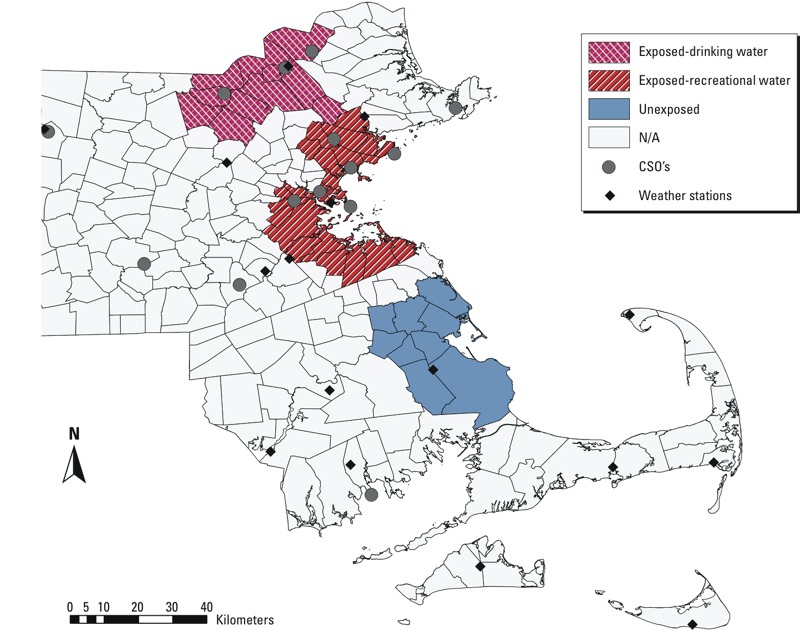
Three exposure classification regions, with classification based on the type of CSO facilities in towns. N/A, not applicable.

*Exposure classification*. Locations of CSO outfalls in Massachusetts were obtained from U.S. EPA Enforcement and Compliance History Online System (U.S. EPA 2014b). We considered three regions for analysis: one with recreational water exposure to CSOs (exposed–recreational water region), one with drinking water exposure to CSOs (exposed–drinking water region), and an area without exposure to CSOs (unexposed region) (Figure 1). In the exposed–recreational water region, CSOs discharge directly into a water body used for recreation, the Boston Harbor. This region included 24 neighboring cities and towns surrounding the harbor likely to recreate in the harbor. Boston Harbor is typically used for sailing, fishing, kayaking, and recreational activities with limited direct water contact. In the exposed–drinking water region, which included 11 neighboring towns, CSOs discharge into river waters used as a drinking water source. Despite being a historically industrial and polluted waterway, the Merrimack River, which serves this region, is the only river in New England from which communities draw drinking water directly (Leo 2014). The unexposed region included 9 neighboring towns that do not have CSO outfalls.

*Exposure data*. Daily rainfall and temperature data were obtained from the National Climatic Data Center (http://www.ncdc.noaa.gov/). One weather station in each exposure region was selected based on completeness of data for the study period 2003–2007. Three time series for each region indicating days with precipitation ≥ 90th, 95th, and 99th percentiles were developed based on the overall distribution for the region, allowing for sensitivity analysis. The 99th percentile was included because we anticipated these events would be severe enough to potentially trigger a CSO event.

Time series of daily average temperature for each exposure region from the same weather stations was included in the models as a potential confounder. Temperature has been associated with environmental transmission of pathogens (Checkley et al. 2000; Fleury et al. 2006; Naumova et al. 2007) and the health of vulnerable subpopulations (Kovats and Hajat 2008).

*ER data*. Daily ER visits were obtained from the State of Massachusetts Division of Health Care Finance and Policy (http://chiamass.gov/) for 2003–2007. Each record represents an ER visit and includes patient information, such as age, sex, town of residence, diagnoses, and admission data and time.

Data for ER visits that included diagnosis for gastrointestinal illness were abstracted from the complete dataset. GI illness was defined by the following ICD-9CM (*International Classification of Diseases, Ninth Revision, Clinical Modification*) codes: 001–009, 558.9, 787.0, 787.01, 787.03, 787.4, 787.9, 787.91. Cases were grouped based on town of residence to create a daily time series of ER visits for each exposure region. Due to the potential for differences in susceptibility according to age, analyses were also stratified by age group (< 5, 5–19, 20–64, and ≥ 65 years.) Daily rates of ER visits for GI illness per 100,000 population were calculated using population estimates for 2005 (U.S. Census Bureau 2011), the mid-point of the study period, for each region and by age group. The use of limited, de-identified admissions records was designated as non-human subjects research and exempt from institutional review board review as determined by the Human Subjects Research Protocol Officer of the U.S. EPA, Office of Research and Development, Research Triangle Park, North Carolina.

Data to describe the towns in each exposure region were abstracted from the 2000 U.S. Census of Population and Housing (U.S. Census Bureau 2013), including population density (population per square mile), median per capita income (dollars), household size (average number of people per household), and septic tank density (number of septic tanks per square mile). Analysis of variance (ANOVA) was used to assess differences in these descriptive factors across exposure regions. An α of 0.05 was considered statistically significant.

*Statistical analysis*. Model estimation. We used a distributed lag Poisson regression model (Schwartz 2000; Zanobetti et al. 2000) to assess associations between heavy precipitation and daily rates of ER visits for GI infections in the three regions. The distributed lag model estimates the cumulative effect of precipitation over the entire lag period following a heavy precipitation event (Gasparrini 2011; Gasparrini et al. 2010). This allows for consideration of various pathogens that cause GI illness but have different incubation periods and has been previously used in studies of turbidity and GI infections (Morris et al. 1998; Naumova et al. 2003). We used a quasi-Poisson distribution (Tjur 1998) to account for overdispersion. The model is given by Equation 1, where *ER* represents the time series of rate of ER visits for GI illness on day *t*, *precip* is the time series indicating days with precipitation greater than the cut-off percentile (90th, 95th, or 99th), *temp* is the time series for daily average temperature, and *ns(t,f)* represents the natural spline function for time used to control for unmeasured covariates. The summation is calculated over the selected number of lag days, *lag*, following the heavy precipitation events. β*_l_* is the lag weight or coefficient placed on *l* days previous to the date of heavy precipitation. We used the finite distribution lag model assuming a maximum number of lag days beyond which heavy precipitation does not affect the rate of ER visits for GI illness. The degrees of freedom (*f* in the natural spline function) was determined based on the minimum residual autocorrelation (Peng et al. 2006).

log[*E*(*ER_t_*)] = α + Σ*^^lag^^_l_*_= 0_ β_l *_ precip_t – l_ + γ _*_ temp_t_ + ns(*t,f*). [1]

For interpretation, the CRR is the cumulative risk of the rate of ER visits for GI for the 8-day period following a day with rainfall over the 99th percentile compared with an 8-day period following days without rainfall. Analyses were conducted in R (R Core Team 2014) and, specifically, the dlnm package for the distributed lag model (Gasparrini 2011).

Lag selection. Previous studies demonstrated that GI illness tends to peak several days after an extreme precipitation event (Schuster et al. 2005; Schwartz et al. 1997, 2000). The incubation period for GI pathogens varies from an average of a few hours, for bacterial pathogens, up to 7 days, for protozoal pathogens (Nelson and Williams 2013). We assessed an 8-day lag to capture the majority of GI illnesses caused by waterborne exposure. We also considered 4-day and 15-day lag periods to evaluate lag times for different pathogens. We utilized a uniform weighted lag structure, assuming that probability of infection would be equal across the lag period.

Seasonal subanalysis. A subanalysis to assess associations between extreme precipitation (≥ 99th percentile) and rate of ER visits for GI illness by season was conducted for each region and age category. Seasons were defined, as in previous studies (Curriero et al. 2001; Nichols et al. 2009), as spring (March, April, May), summer (June, July, August), fall (September, October, November), and winter (December, January, February).

## Results

**Figure 2:**
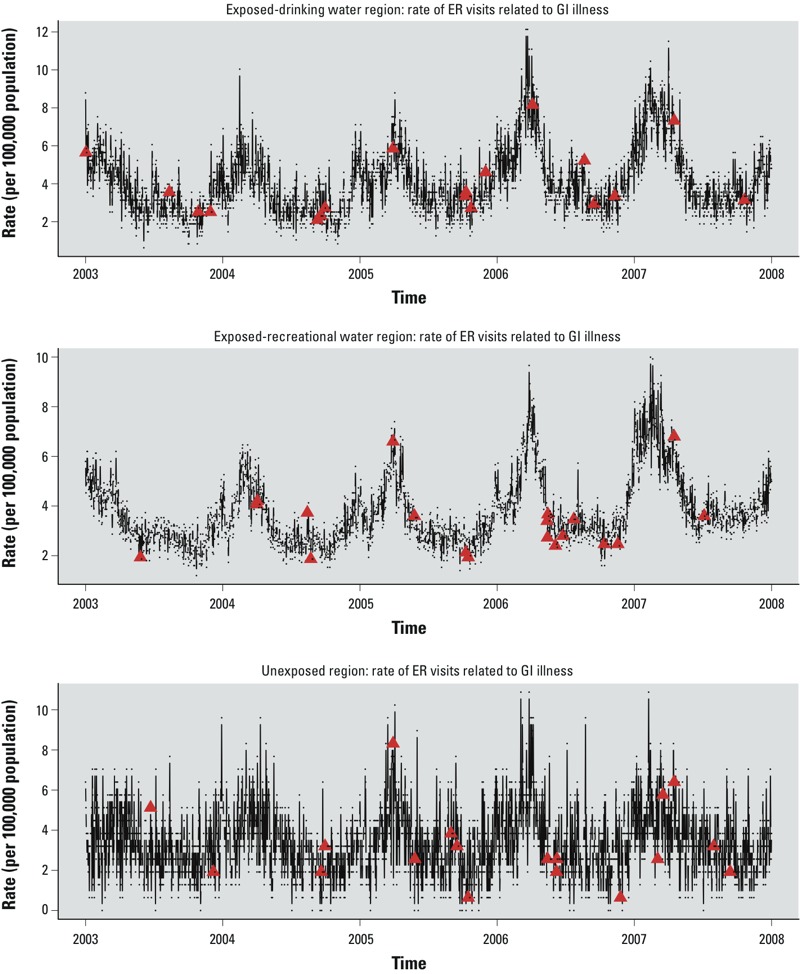
Time series of rate of daily ER visits for GI illness for each exposure region (per 100,000 population) indicated by dots, with days of with extreme precipitation (≥ 99th percentile) indicated by triangles.

*Descriptive analysis*. The three regions experienced similar rainfall patterns over the 5-year (1,826 days) study period. The exposed–drinking water region had 999 days of rain (55% of days), ranging from 0.005 to 6.76 in. The 90th, 95th, and 99th percentiles for rainfall were 0.41, 0.64, and 1.33 in, respectively. The exposed–recreational water region had 848 days of rainfall (46% of days), ranging from 0.005 to 4.32 in. The 90th, 95th, and 99th percentiles for rainfall were 0.37, 0.70, and 1.60 in, respectively. The unexposed region had 928 days of rainfall (51% of days), ranging from 0.005 to 3.61 in. The 90th, 95th, and 99th percentiles for rainfall were 0.39, 0.77, and 1.97 in, respectively. Time series for daily rate of ER visits by region with indication of days of extreme precipitation (≥ 99th percentile) are shown in Figure 2.

The three regions varied by size and characteristics. As we expected, the most urban of the three regions, the exposed–recreational water region that includes the Boston metropolitan area, had the highest population, population density, and median per capita income and the lowest average household size and septic tank density (Table 1).

**Table 1:** Descriptive statistics for each of the three exposure regions, including number of cities and towns, total population (2005), total population by age group (2005), total number of ER visits for GI illness, number of daily ER visits for GI illness, and daily rate of ER visits for GI illness per 100,000 population, as well as sociodemographic factors, including average household size, population density, septic tank density, and median per capita income.

Characteristic	Exposed–drinking water region	Exposed–recreational water region	Unexposed region	*p*-Value^*a*^
Cities/towns	11	24	9
Total population^*b*^	478,071	1,501,163	156,264
Age group
< 5 years	35,800	83,649	10,733
5–19 years	108,670	267,468	30,684
20–64 years	277,049	960,827	92,902
≥ 65 years	56,552	189,218	21,945
Average daily ER visits for GI illness	19.37 ± 2.93	56.01 ± 9.60	5.25 ± 0.29
Average daily rate of ER visits for GI illness(per 100,000 population)	4.05 ± 0.62	3.73 ± 0.61	3.36 ± 0.16
Population density (population/mile^2^)^*c*^	2732.17 ± 3177.53	5983.30 ± 5133.24	552.64 ± 214.94	0.004
Median per capita income (dollars)^*c*^	24,953 ± 8,951	27,113 ± 7,660	23,738 ± 5,787	0.27
Average household size (*n*)^*c*^	2.72 ± 0.13	2.47 ± 0.22	2.84 ± 0.15	< 0.0001
Septic tank density (tanks/mile^2^)^*c*^	0.05 ± 0.04	0.02 ± 0.04	0.14 ± 0.03	< 0.0001
Abbreviations: ER, emergency room; GI, gastrointestinal. Values shown are number or mean ± SD. ^***a***^Differences across groups assessed using ANOVA. ^***b***^Population estimates for 2005 from U.S. Census Bureau (2011). ^***c***^Sociodemographic data from U.S. Census 2000 (U.S. Census Bureau 2013).				

*Model results*. The distributed lag models demonstrated that the association between rainfall events and rate of ER visits for GI illness differed by the presence and type of CSOs only when the 99th percentile was used as the cutoff. The 90th and 95th percentile cutoffs for precipitation did not demonstrate significant positive associations with the rate of ER visits for GI illness in any region (Table 2). In the exposed–drinking water region, there was a significantly increased cumulative risk for rate of ER visits for GI illness for all ages 8 days following a 99th percentile rainfall event, CRR: 1.13 [95% confidence interval (CI): 1.00, 1.28], after controlling for daily average temperature and time trends (Table 2). This suggests a 13% increase in the expected rate of ER visits over the 8-day period following an extreme precipitation event compared with no precipitation in the region with CSO outfalls to receiving waters that are a source of drinking water. In the exposed–recreational water region and the unexposed region, there was no association between rate of ER visits for GI and extreme precipitation events [CRR: 0.95 (95% CI: 0.88, 1.03) and CRR: 1.05 (95% CI: 0.84, 1.32), respectively].

**Table 2:** Cumulative risk ratios of rate of emergency room visits for gastrointestinal illness associated with precipitation (≥ 90th, 95th, and 99th percentile) by exposure region and age group for all three regions as estimated by distributed lag model with an 8-day lag.

Age group	No. of ER visits	Cumulative RR^*a*^ (95% CI)		
		90th percentile	95th percentile	99th percentile
Exposed–drinking water region
All ages	35,358	0.97 (0.93, 1.01)	0.95 (0.89, 1.01)	1.13 (1.00, 1.28)
< 5 years	8,526	0.98 (0.91, 1.07)	0.95 (0.85, 1.07)	1.19 (0.95, 1.49)
5–19 years	6,267	1.02 (0.91, 1.14)	1.02 (0.88, 1.19)	1.23 (0.89, 1.69)
20–64 years	17,577	0.95 (0.89, 0.99)	0.94 (0.87, 1.01)	1.08 (0.91, 1.27)
≥ 65 years	2,988	0.97 (0.86, 1.11)	0.98 (0.82, 1.18)	1.32 (0.92, 1.88)
Exposed–recreational water region
All ages	102,272	0.99 (0.97, 1.02)	0.98 (0.95, 1.02)	0.95 (0.88, 1.03)
< 5 years	25,197	0.97 (0.92, 1.03)	0.98 (0.91,1.05)	0.92 (0.78, 1.09)
5–19 years	15,613	0.92 (0.86, 0.98)	0.94 (0.85, 1.04)	0.93 (0.74, 1.15)
20–64 years	53,091	1.00 (0.97, 1.04)	0.98 (0.93, 1.02)	0.95 (0.86, 1.06)
≥ 65 years	8,371	1.06 (0.98, 1.14)	1.03 (0.93, 1.14)	0.94 (0.74, 1.20)
Unexposed region
All ages	9,584	1.02 (0.95, 1.10)	1.05 (0.94, 1.16)	1.05 (0.84, 1.32)
< 5 years	1,834	0.99 (0.85, 1.17)	1.04 (0.84, 1.30)	0.54 (0.28, 1.03)
5–19 years	1,774	1.01 (0.84, 1.21)	1.02 (0.79, 1.31)	0.95 (0.53, 1.68)
20–64 years	5,160	1.02 (0.92, 1.11)	1.00 (0.87, 1.15)	1.20 (0.91, 1.59)
≥ 65 years	816	0.95 (0.75, 1.21)	1.13 (0.82, 1.55)	1.31 (0.71, 2.42)
Abbreviations: ER, emergency room; GI, gastrointestinal. ^***a***^Model estimates are adjusted for daily average temperature and time trends.				

Positive associations were observed for all age categories in the exposed–drinking water region. The largest estimated increase, although not statistically significant, was among residents ≥ 65 years of age, CRR: 1.32 (95% CI: 0.92, 1.88). Strong associations were also seen in children (5–19 years), CRR: 1.23 (95% CI: 0.89, 1.69). The exposed–recreational water region did not demonstrate positive associations for any age category. The unexposed region demonstrated positive associations in the adult (20–64 years) and elderly (≥ 65 years) age categories, CRR: 1.20 (95% CI: 0.91, 1.59) and CRR: 1.31 (95% CI: 0.71, 2.42), respectively.

The results and trends for rainfall ≥ 99th percentile and GI illness using 4-day and 15-day lag periods were similar to model estimates for the 8-day lag (see Supplemental Material, Table S1). The consistent positive associations estimated for the exposed– drinking water region suggest an increase in ER visits during the first 4 days that may continue for up to 15 days after extreme rainfall events.

## Discussion

We found evidence of an association between the rate of ER visits for GI illness and heavy precipitation that appeared to be limited to the region with CSO outfalls to drinking water sources. Specifically, we estimated that, compared with no precipitation, extreme precipitation events (≥ 99th percentile) were associated with a 13% increase in the expected rate of ER visits for GI illness in this region over an 8-day period for all age groups combined. In the region with CSO outfalls to recreational waters and the region without exposure to CSO outfalls, there were no associations between extreme rainfall events and rate of ER visits for GI illness. Our findings suggest that extreme precipitation events may trigger CSO events that affect local drinking water quality in some areas. Our study is the first that we know of to demonstrate an association between extreme precipitation and GI illness that differs according to the presence of CSOs and whether the receiving waters are used for recreation or as a source of drinking water.

Contrary to previous findings (Bush et al. 2014; Carlton et al. 2014; Drayna et al. 2010; Nichols et al. 2009; Curriero et al. 2001), heavy rainfall events (≥ 90th and 95th percentile) were not associated with increased rates of ER visits for GI illness in any of the three exposure regions. Drinking water turbidity, often a result of heavy precipitation, was positively associated with hospital admissions for children between 0 and 15 years of age in Philadelphia at 8- and 13-day lags (Schwartz et al. 1997). An increase in interquartile range of turbidity was associated with a 1.47 increase in relative risk of self-reported GI infections in the town of Cherepovets, Russia (Egorov et al. 2003). In contrast, a study of eight drinking water treatment plants in Atlanta, Georgia, demonstrated no association between filtered water turbidity and emergency department visits for GI illness (Tinker et al. 2010). Rainfall events greater than the 93rd percentile were associated with a 2.28 times (95% CI: 1.22, 4.23) increased risk for waterborne outbreaks in Canada (Thomas et al. 2006). A study conducted in the United Kingdom estimated that laboratory-confirmed cryptosporidiosis increased by 27% (95% CI: 21%, 33%) when the cumulative rainfall for the prior week exceeded the 75th percentile, or 0.86 in. (Naumova et al. 2005). A study of pediatric emergency room visits for acute GI illness found that any rainfall 4 days prior was significantly associated with an 11% increase in ER visits for GI illness (Drayna et al. 2010). A recent study conducted in Chennai, India, estimated an increased cumulative risk ratio for hospital admissions for GI illness for the 15-day period following an extreme precipitation event (≥ 90th percentile) was 1.60 (95% CI: 1.29, 1.98) (Bush et al. 2014). Previous work on the effect of precipitation on GI illness has also demonstrated stronger associations in the most vulnerable age subpopulations, children and the elderly (Bush et al. 2014; Drayna et al. 2010; Redman et al. 2007). For the exposed–drinking water region, our age-stratified results demonstrated associations in children < 5 years of age, children of 5–19, and the elderly (≥ 65 years) that were stronger than the association in the adult (20–64 years) group.

Few studies have considered the impact of CSO events on human enteric infection. Using a risk assessment approach, one study estimated that the risk of contracting GI illness from incidental ingestion of water affected by CSO outfalls was 0.14 and nearly 0.70 over the course of a year for visitors and recreators, respectively (Donovan et al. 2008). Our findings in the exposed–drinking water region are consistent with the findings of Redman et al. (2007), who found that pediatric ER visits for diarrheal illness increased 3–7 days following sewage bypass events for those using drinking water from Lake Michigan. Another study in Wisconsin also demonstrated associations between a winter sewage release event and increased pediatric visits for GI illness (Drayna et al. 2010). Although CSOs are known to adversely impact source water quality and introduce pathogens (Donovan et al. 2008; Marsalek and Rochfort 2004; McLellan and Salmore 2003), we did not confirm that municipal water supplies in the exposed–drinking water region were adversely impacted during the study period.

Selection of an appropriate lag period and structure for the studies of waterborne pathogens must consider expected time due to pathogen transport in the environment, pathogen incubation periods, and time to seek care after illness begins (Egorov et al. 2003). In addition, environmental pathogen transport can vary significantly based on the route of exposure, either drinking water or recreational water. In previous studies of associations between drinking water turbidity and GI illness, authors concluded that a Poisson or gamma distribution and lag structures with a mean of 7–8 days were appropriate for estimation of distributed effects (Egorov et al. 2003; Naumova and MacNeill 2008). The lag periods considered in our study—4, 8, and 15 days—are comparable to previous studies (Bush et al. 2014; Drayna et al. 2010; Egorov et al. 2003; Schwartz et al. 1997). However we assumed a uniform weighted lag structure and the use of a Poisson or gamma distribution lag structure may provide more accurate estimation of cumulative risk.

We did not observe an association between heavy precipitation and rate of ER visits for GI in areas with recreational water impacted by CSOs. This lack of an association may be because recreational water contact is likely to be highly localized and seasonal. Although a recent study of triathletes found a significantly increased risk for swimming in contaminated waters (Harder-Lauridsen et al. 2013), the majority of recreational activities in our study area (Boston Harbor) would involve only limited water contact; therefore, the amount of water ingested may not be sufficient to cause an observable increase in rate of ER visits for GI illness (Dorevitch et al. 2011, 2012). Previous studies have demonstrated effect modification by season (Bush et al. 2014; Carlton et al. 2014; Drayna et al. 2010; Nichols et al. 2009), but our seasonality analysis did not demonstrate associations between extreme rainfall and rate of ER visits for GI illness in any of the three regions, suggesting that increased seasonal recreational exposure is not associated with increases in rates of ER visits for GI illness (see Supplemental Material, Table S2). Also, contrary to previous findings, there was no association between extreme precipitation in regions without a CSO, potentially due to the lower concentrations of untreated sewage discharged to water bodies.

The three exposure regions considered were heterogeneous and differed significantly from each other, which is a study limitation. Previous studies reported that the confounders and risk factors for enteric infections in Massachusetts were geographically distributed. The use of mixed surface and ground drinking water supplies (Naumova et al. 2000) and high septic tank density (Cohen et al. 2008), both of which are geographically distributed, have been associated with elevated risk for enteric infections in Massachusetts. Differences in these factors among our exposure regions may explain some of the differences in associations between extreme precipitation and rates of ER visits for GI illness among the three regions. However, due to the nature of CSOs, which are typically built in older urban areas, we are unable to define more comparable exposure regions to explore this association.

With climate change, it is predicted that extreme rainfall events will increase and therefore increase the likelihood of CSO events (Patz et al. 2008). In the United States, it has been observed that since 1990 a large percentage of precipitation has come in the form of intense single day events (U.S. EPA 2014a). In the Northeast, single day heavy rainfall events are expected to increase, and the 99th percentile of rainfall events has increased by more than 1 in. for most of the region (Spierre and Wake 2010). The increase in intensity of precipitation events will trigger more CSO events because these systems are not designed to handle large volumes of water. Increases in overflow events will put more water systems, both drinking and recreational water, at an increased risk for pathogen contamination. CSO systems are common in densely populated urban areas in the United States, such as New England and the Great Lakes area; therefore, these events can put large populations at risk for increased GI illness. Although communities are expected to develop long-term CSO control plans (U.S. EPA 1995), these plan can be expensive and require significant infrastructure changes; therefore, it is useful to understand the health impact of these events.

Although we observed an association between extreme precipitation and rate of ER visits for GI illness in the exposed–drinking water region, we lacked detailed data to confirm the drinking water sources were impacted by the severe precipitation events and mediated by a CSO. This analysis would, ideally, be conducted using dates of CSO events; however, these data were not available for this analysis. The ER data are collected for administrative purposes and do not provide individual-level information on behaviors, such as drinking water source and recreational water exposures, which could be used to adjust for in this analysis. Studies have demonstrated that GI illness is underreported and that only a portion of actual cases are recognized (Craun et al. 2010; Schuster et al. 2005; Yoder et al. 2008). In addition, ER visits for GI illness represent only a fraction of all GI illnesses in a community and may not be representative of GI illness occurring in the community (Mead et al. 1999).

## Conclusions

We demonstrated that the association between extreme rainfall and ER visits for GI diseases differed by the presence and type of CSOs. Only in the region with CSO outfalls to drinking water sources did we find a significant increase in the expected rate of ER visits for GI illness for all ages in the 8-day period following an extreme precipitation event after controlling for daily average temperature and time trends. In light of expected increases in extreme precipitation events, our findings suggest that drinking water quality may be adversely impacted by the presence of CSOs that discharge into drinking water sources after heavy rainfall.

## Supplemental Material

(119 KB) PDF
