# Supplementary material for: Extreme Precipitation and Emergency Room Visits for Gastrointestinal Illness in Areas with and without Combined Sewer Systems: An Analysis of Massachusetts Data, 2003–2007
Source: Environ Health Perspect. 2015 Apr 9;123(9):873–9. doi: 10.1289/ehp.1408971 (PMC4559956; doi:10.1289/ehp.1408971)
Supplement: (119 KB) PDF [file ehp.1408971.s001.acco.pdf]

Environ Health Perspect
doi: 10.1289/ehp.1408971

Note to Readers: *EHP* strives to ensure that all journal content is accessible to all readers. However, some figures and Supplemental Material published in *EHP* articles may not conform to 508 standards due to the complexity of the information being presented. If you need assistance accessing journal content, please contact ehp508@niehs.nih.gov. Our staff will work with you to assess and meet your accessibility needs within 3 working days.

Supplemental Material

Extreme Precipitation and Emergency Room Visits for Gastrointestinal Illness in Areas with and without Combined Sewer Systems: An Analysis of Massachusetts Data, 2003–2007

Jyotsna S. Jagai, Quanlin Li, Shiliang Wang, Kyle P. Messier, Timothy J. Wade, and Elizabeth D. Hilborn

Table of Contents

Table S1. Cumulative risk ratios of emergency room visits for gastrointestinal illness associated with extreme precipitation (≥ 99th percentile) by exposure region and age group for all three regions as estimated by distributed lag model with an 8-day lag, 4-day lag, and 15-day lag.

Table S2. Cumulative risk ratios of emergency room visits for gastrointestinal illness associated with extreme precipitation (≥ 99th percentile) by exposure region, age group and season for all three regions as estimated by distributed lag model with an 8-day lag.

Table S1. Cumulative risk ratios of emergency room visits for gastrointestinal illness associated with extreme precipitation (≥ 99th percentile) by exposure region and age group for all three regions as estimated by distributed lag model with an 8-day lag, 4-day lag, and 15-day lag.

	8 Day Lag	4 Day Lag	15 Day Lag
**Age Group**	**Cumulative RR (95% CI)**	**Cumulative RR (95% CI)**	**Cumulative RR (95% CI)**
**Exposed – Drinking Water Region**			
All ages	1.13 (1.00, 1.28)	1.13 (1.00, 1.28)	1.13 (1.00, 1.28)
≤ 5 years	1.19 (0.95, 1.49)	1.22 (0.97, 1.53)	1.21 (0.97, 1.52)
6 – 18 years	1.23 (0.89, 1.69)	1.22 (0.88, 1.69)	1.22 (0.88, 1.69)
19 – 64 years	1.08 (0.91, 1.27)	1.08 (0.92, 1.28)	1.08 (0.92, 1.28)
≥ 65 years	1.32 (0.92, 1.88)	1.31 (0.92, 1.87)	1.32 (0.92, 1.89)
**Exposed – Recreational Water Region**			
All ages	0.95 (0.88, 1.03)	0.95 (0.88, 1.03)	0.95 (0.88, 1.03)
≤ 5 years	0.92 (0.78, 1.09)	0.94 (0.79, 1.10)	0.92 (0.78, 1.09)
6 – 18 years	0.93 (0.74, 1.15)	0.95 (0.76, 1.18)	0.93 (0.74, 1.15)
19 – 64 years	0.95 (0.86, 1.06)	0.96 (0.87, 1.07)	0.95 (0.86, 1.06)
≥ 65 years	0.94 (0.74, 1.20)	0.94 (0.74, 1.19)	0.94 (0.74, 1.20)
**Unexposed Region**			
All ages	1.05 (0.84, 1.32)	1.05 (0.84, 1.32)	1.05 (0.84, 1.32)
≤ 5 years	0.54 (0.28, 1.03)	0.54 (0.29, 1.06)	0.54 (0.28, 1.03)
6 – 18 years	0.95 (0.53, 1.68)	0.93 (0.52, 1.68)	0.93 (0.52, 1.68)
19 – 64 years	1.20 (0.91, 1.59)	1.20 (0.91, 1.59)	1.20 (0.91, 1.60)
≥ 65 years	1.31 (0.71, 2.42)	1.30 (0.70, 2.39)	1.31 (0.71, 2.42)

Table S2. Cumulative risk ratios of emergency room visits for gastrointestinal illness associated with extreme precipitation (≥ 99th percentile) by exposure region, age group and season for all three regions as estimated by distributed lag model with an 8-day lag.

	**Spring[a]**	**Summer[a]**	**Fall[a]**	**Winter[a]**
**Age Group**	**Cumulative RR (95% CI)**	**Cumulative RR (95% CI)**	**Cumulative RR (95% CI)**	**Cumulative RR (95% CI)**
**Exposed – Drinking Water Region**				
All ages	0.73 (0.58, 0.91)	0.94 (0.72, 1.24)	0.96 (0.73, 1.25)	1.15 (0.93, 1.42)
≤ 5 years	0.57 (0.37, 0.88)	1.30 (0.75, 2.26)	0.77 (0.41, 1.43)	0.97 (0.64, 1.46)
6 – 18 years	0.71 (0.42, 1.21)	1.02 (0.55, 1.90)	1.14 (0.60, 2.14)	1.40 (0.96, 2.06)
19 – 64 years	0.83 (0.63, 1.10)	0.87 (0.61, 1.24)	0.93 (0.67, 1.31)	1.08 (0.80, 1.47)
≥ 65 years	0.84 (0.43, 1.62)	0.66 (0.27, 1.61)	1.25 (0.59, 2.63)	1.27 (0.66, 2.47)
**Exposed – Recreational Water Region**				
All ages	1.06 (0.92, 1.22)	1.09 (0.93, 1.26)	0.98 (0.83, 1.15)	1.05 (0.88, 1.24)
≤ 5 years	1.18 (0.92, 1.51)	0.99 (0.70, 1.41)	0.83 (0.55, 1.24)	1.13 (0.84, 1.52)
6 – 18 years	0.89 (0.63, 1.26)	1.38 (0.97, 1.95)	1.01 (0.70, 1.45)	1.23 (0.91, 1.66)
19 – 64 years	0.98 (0.83, 1.16)	1.12 (0.93, 1.35)	0.98 (0.78, 1.23)	0.93 (0.74, 1.16)
≥ 65 years	1.46 (1.03, 2.08)	0.72 (0.44, 1.18)	1.32 (0.85, 2.05)	1.04 (0.66, 1.64)
**Unexposed Region**				
All ages	1.06 (0.74, 1.51)	0.48 (0.24, 0.95)	0.75 (0.44, 1.28)	0.83 (0.54, 1.27)
≤ 5 years	0.94 (0.46, 1.92)	0.77 (0.18, 3.20)	1.56 (0.22, 10.80)	0.38 (0.11, 1.34)
6 – 18 years	1.29 (0.62, 2.69)	0.39 (0.06, 2.49)	0.64 (0.15, 2.72)	0.93 (0.37, 2.32)
19 – 64 years	0.99 (0.59, 1.67)	0.47 (0.19, 1.14)	0.29 (0.10, 0.81)	1.05 (0.60, 1.82)
≥ 65 years	0.42 (--, --[b])	0.30 (0.04, 2.15)	2.85 (0.05, 165.78)	1.67 (0.00, 1108.89)

[a]Defined as Spring (March, April, May), Summer (June, July, August), Fall (September, October, November), Winter (December, January, February). [b]Did not converge.
